# Epidemiology of Pathogen-Specific Respiratory Infections among Three US Populations

**DOI:** 10.1371/journal.pone.0114871

**Published:** 2014-12-30

**Authors:** Jennifer M. Radin, Anthony W. Hawksworth, Peter E. Kammerer, Melinda Balansay, Rema Raman, Suzanne P. Lindsay, Gary T. Brice

**Affiliations:** 1 Operational Infectious Diseases Department, Naval Health Research Center San Diego, San Diego, California, United States of America; 2 Joint Doctoral Program in Public Health (Epidemiology), San Diego State University/University of California San Diego, San Diego, California, United States of America; 3 Department of Family and Preventive Medicine, University of California San Diego, La Jolla, California, United States of America; 4 Graduate School of Public Health, San Diego State University, San Diego, California, United States of America; Centers for Disease Control and Prevention, United States of America

## Abstract

**Background:**

Diagnostic tests for respiratory infections can be costly and time-consuming. Improved characterization of specific respiratory pathogens by identifying frequent signs, symptoms and demographic characteristics, along with improving our understanding of coinfection rates and seasonality, may improve treatment and prevention measures.

**Methods:**

Febrile respiratory illness (FRI) and severe acute respiratory infection (SARI) surveillance was conducted from October 2011 through March 2013 among three US populations: civilians near the US–Mexico border, Department of Defense (DoD) beneficiaries, and military recruits. Clinical and demographic questionnaire data and respiratory swabs were collected from participants, tested by PCR for nine different respiratory pathogens and summarized. Age stratified characteristics of civilians positive for influenza and recruits positive for rhinovirus were compared to other and no/unknown pathogen. Seasonality and coinfection rates were also described.

**Results:**

A total of 1444 patients met the FRI or SARI case definition and were enrolled in this study. Influenza signs and symptoms varied across age groups of civilians. Recruits with rhinovirus had higher percentages of pneumonia, cough, shortness of breath, congestion, cough, less fever and longer time to seeking care and were more likely to be male compared to those in the no/unknown pathogen group. Coinfections were found in 6% of all FRI/SARI cases tested and were most frequently seen among children and with rhinovirus infections. Clear seasonal trends were identified for influenza, rhinovirus, and respiratory syncytial virus.

**Conclusions:**

The age-stratified clinical characteristics associated with influenza suggest that age-specific case definitions may improve influenza surveillance and identification. Improving identification of rhinoviruses, the most frequent respiratory infection among recruits, may be useful for separating out contagious individuals, especially when larger outbreaks occur. Overall, describing the epidemiology of pathogen specific respiratory diseases can help improve clinical diagnoses, establish baselines of infection, identify outbreaks, and help prioritize the development of new vaccines and treatments.

## Introduction

Acute respiratory infections make up a huge proportion of disease burden in the United States and globally, with an estimated 94 037 000 disability adjusted life years and 3.9 million deaths worldwide each year [1]. Respiratory infections are often difficult to diagnose clinically due to nonspecific and overlapping symptoms. Additionally, diagnostic tests can be time-consuming and costly and often require trained and well-equipped laboratories, making laboratory confirmation of each case impractical. However, laboratory results from various surveillance populations can be paired with clinical, demographic, and seasonality variables to create models that can give timely predictions of disease outcomes. Preventive measures and treatments to reduce respiratory disease burden can also be improved through routine surveillance by gaining a better understanding of the percent positivity of pathogens among acute respiratory cases, seasonality, and coinfection occurrence.

Currently, limited respiratory disease etiology studies have been done in the United States [2], [3], [4], despite many being done in other countries [5], [6], [7], [8], [9], [10], [11], [12], [13], [14]. Additionally, few viral etiology studies have collected clinical signs and symptoms and assessed their association with a broad range of respiratory pathogens [9], [13], [14]. Most descriptive studies and predictive models for respiratory diseases have focused on identifying influenza using clinical signs and symptoms [15], [16], [17], [18], [19], [20], however few have been age-stratified and therefore may have missed some important differences in clinical presentation by age. Understanding US-specific disease burden and seasonality is important, since disease incidence, distribution, and seasonality may vary between populations, regions, and climates. This study aimed to describe characteristics associated with specific respiratory pathogens, as well as the etiology, seasonality, and coinfection rates among three US populations: military recruits, Department of Defense (DoD) beneficiaries, and civilians living near the US–Mexico border.

The results of this study can help improve timely and more accurate diagnosis, inform treatment plans, establish baselines of infection, identify outbreaks, and help prioritize the development of new vaccines and future treatments.

## Methods

### Participants

FRI and SARI surveillance was conducted between October 2011 and March 2013 among three surveillance groups in the United States: civilians near the US–Mexico border, DoD dependents, and military recruits. Separate seasonality data from the same populations was available for January 2012 through December 2013. Recruits are typically young and healthy adults who enter into an 8–12 week “boot camp” training program, which involves strenuous, and physically demanding activities and living in high-density barracks. During the first week of training, recruits receive a series of vaccinations, including influenza (seasonally) and adenovirus. Most training centers also administer at least one dose of bicillin to incoming trainees as prophylaxis against *Streptococcus* bacteria. The DoD dependent population is made up of the families of active duty and retired military personnel. This population consists of all ages and has good access to health care through the TRICARE health care program. The border sites are not associated with the military and typically capture underserved populations living near the US–Mexico border in California and Arizona. The border sites are part of a broader Border Infectious Disease surveillance program run by the US Centers for Disease Control and Prevention and San Diego and Imperial counties, for which the Naval Health Research Center (NHRC) serves as the testing laboratory.

The FRI recruit sites included Marine Corps Recruit Depot (MCRD) San Diego, California; Air Force Basic Military Training center, Lackland Air Force Base, Texas; Naval Training Command, Great Lakes, Illinois; US Army Training Center (USATC) Fort Leonard Wood, Missouri; USATC Fort Jackson, South Carolina; USATC Fort Benning, Georgia; MCRD Parris Island, South Carolina; and Coast Guard Training Center, Cape May, New Jersey. The DoD beneficiaries sites included Naval Medical Center San Diego, California; TRICARE Clairemont Mesa, San Diego, California; and TRICARE Great Lakes, Illinois. FRI and SARI border sites included clinics and hospitals in San Ysidro, California; Calexico, California; Brawley, California; El Centro, California; Chula Vista, California; Selles, Arizona; and Tucson, Arizona.

### Ethics Statement

This research was conducted in compliance with all applicable federal and international regulations governing the protection of human subjects in research. The research conducted in the recruit and DoD beneficiary populations underwent NHRC IRB approval (NHRC protocols 31230 and NHRC.1999.0002) and written consent or parental guardian consent for minors was obtained for all participants. The data collected from the border population was part of a surveillance program run by the US Centers for Disease Control and Prevention (CDC) and was considered non-research by the NHRC IRB. NHRC staff members only provided diagnostic support and only received non-personally identifiable data.

### Procedures

Case definitions were slightly different for each of the three populations. In the recruit population, an FRI case was defined as a person who sought medical care and had an oral temperature ≥38.0°C (100.5°F) and either cough or sore throat. For the beneficiary population and border populations, the same FRI case definition was used; however, a fever ≥37.8°C (100.0°F) or subjective fever was used. Additionally, inpatients who met SARI case definition at select border sites were also sampled. The SARI case definition included people who presented with either fever ≥37.8°C (100.0°F) or feeling feverish/chills, in addition to cough, and hospital admission, with onset in the last 10 days. Additionally, children under age five were included if they were admitted to the hospital with clinical suspicion of pneumonia.

Nasal or combination nasal/pharyngeal swabs and questionnaire data were collected from a convenience sample of up to 20 patients per week per site who sought medical attention, met the FRI or SARI case definition, and provided written informed consent. Specimens were placed in universal transport medium [21], preserved at −80°C, and later transported on dry ice to the reference laboratory at the NHRC every one to two weeks for testing. Additionally, the following demographic and clinical signs and symptoms were collected from each FRI and SARI case: sex, age, study population, month of illness, pneumonia, sore throat, cough, nausea, shortness of breath, congestion, pink eye, body ache, headache, temperature, number of days of symptoms before seeking care, and date of seeking care.

Samples were extracted using the Roche MagNA Pure LC extraction system following manufacturer’s instructions. Samples were then tested for a broad-spectrum panel consisting of standard PCR gel tests: hMPV (single plex), CoVNL63 (single plex), and CoV229E and CoVOC43 (multiplex). Real-time polymerase chain reaction (PCR) assays were run on the Applied Biosystems 7500 Fast Real-Time PCR System testing for influenza A, influenza B, adenovirus, RSV, rhinovirus, and a bacterial multiplex consisting of *M. pneumoniae*, *C. pneumoniae,* and *B. pertussis*. The rhinovirus primers used in our study have been found to correctly identify 87 of the 100 distinct rhinovirus subtypes and to accurately distinguish rhinoviruses from enteroviruses [22]. All PCR tests were done using in-house primers, except for the influenza A and influenza B which were done using CDC primers. Additionally, viral culture testing for parainfluenza was done for a systematic sample of specimens not positive for influenza or adenovirus.

### Statistical Analysis

The frequencies and means of clinical and demographic characteristics across participants positive for each of the six viruses, bacterial infections, co-infections and no/unknown pathogen were compared. (Table 1). Additionally, characteristics of influenza, other pathogen, or no/unknown pathogen were compared among age stratified groups of DoD beneficiaries and US-Mexico border populations (Table 2) and characteristics of rhinovirus, other pathogen, and no/unknown pathogen were compared among military recruits (Table 3). Chi-square tests for categorical variables and analysis of variance (ANOVA) tests for continuous variables were used to identify signs, symptoms, associated with each pathogen group from all populations. Variables that were univariately associated with the pathogens (*p*<0.15) were investigated further in a multinomial logistic regression model. Variables with a *p*<0.05 were considered in the final adjusted model (Tables S1–S2 in S1 File). Coinfections were coded as “other pathogen” even if one pathogen was influenza or rhinovirus.

**Table 1:** Descriptive Characteristics by All Pathogens, October 2011–March 2013 (n = 1444).

Pathogen(% positivity)	Rhinovirus (16)	Influenza (14)	RSV (6)	Adenovirus (2)	Coronavirus (5)	Bacterial (2)	hMPV (1)	Coinfection (6)	No/UnknownPathogen(n = 703)	Total(n = 1444)
Characteristic	N (%)	N (%)	N (%)	N (%)	N (%)	N (%)	N (%)	N (%)	N (%)	N (%)
Sex (% male)	180 (77)	100 (51)	40 (48)	24 (73)	46 (70)	24 (80)	8 (80)	52 (65)	418 (60)	892 (63)
Age, years										
0–4	24 (10)	38 (20)	57 (71)	8 (24)	12 (8)	1 (3)	5 (50)	30 (38)	94 (14)	270 (19)
5–24	176 (76)	106 (55)	15 (19)	20 (61)	36 (55)	28 (90)	3 (30)	41 (53)	398 (58)	823 (58)
25–49	27 (12)	25 (13)	1 (1)	4 (12)	9 (14)	1 (3)	0 (0)	4 (5)	89 (13)	160 (11)
50+	5 (2)	24 (12)	7 (9)	1 (3)	8 (12)	1 (3)	2 (20)	3 (4)	103 (15)	154 (11)
Studypopulation										
US–Mexico border	29 (12)	87 (44)	41 (48)	13 (39)	14 (21)	4 (13)	5 (45)	24 (30)	189 (27)	406 (28)
DoDbeneficiary	34 (14)	81 (41)	41 (48)	5 (15)	17 (26)	4 (13)	3 (27)	18 (22)	220 (31)	423 (29)
Militaryrecruit	173 (73)	29 (15)	3 (4)	15 (45)	35 (53)	24 (75)	3 (27)	39 (48)	294 (42)	615 (43)
Severity										
Outpatient (FRI)	225 (95)	177 (90)	61 (72)	27 (82)	60 (91)	28 (88)	8 (73)	74 (91)	591 (84)	1251 (87)
Inpatient (SARI)*	11 (5)	20 (10)	24 (28)	6 (18)	6 (9)	4 (13)	3 (27)	7 (9)	112 (16)	193 (13)
Signs andSymptoms(% yes)										
Pneumonia	102 (44)	18 (10)	17 (22)	10 (30)	16 (26)	11 (39)	0 (0)	20 (26)	186 (28)	380 (28)
Sore throat	178 (76)	152 (77)	36 (43)	20 (61)	54 (82)	21 (66)	4 (40)	45 (56)	480 (69)	990 (69)
Cough	218 (93)	192 (97)	83 (100)	30 (91)	63 (95)	30 (94)	9 (90)	79 (98)	590 (84)	1294 (90)
Nausea	92 (39)	87 (45)	26 (32)	7 (22)	18 (27)	11 (34)	4 (40)	23 (29)	256 (37)	524 (37)
SOB	127 (54)	63 (32)	31 (38)	9 (27)	15 (23)	13 (41)	5 (50)	28 (35)	298 (43)	589 (41)
Congestion	203 (87)	154 (79)	56 (70)	19 (58)	55 (83)	20 (65)	8 (80)	56 (71)	468 (69)	1039 (74)
Pink eye	16 (7)	19 (10)	6 (8)	1 (3)	2 (3)	2 (7)	2 (20)	2 (3)	28 (4)	78 (6)
Body ache	123 (53)	125 (64)	28 (34)	12 (36)	39 (59)	21 (66)	3 (30)	37 (46)	405 (58)	793 (55)
Headache	153 (65)	127 (65)	19 (23)	14 (42)	41 (62)	23 (74)	3 (30)	44 (54)	444 (64)	868 (61)
Fever(≥100°C)	100 (42)	137 (70)	52 (61)	21 (64)	36 (55)	21 (66)	8 (73)	47 (58)	355 (51)	777 (54)
Temperature(°F), mean (SD)	99.5 (1.8)	100.9 (1.9)	100.6 (2.1)	100.7 (2.1)	100.0 (1.9)	100.6 (2.0)	101.3 (1.6)	100.2 (1.9)	99.9 (2.0)	100.1 (2.0)
Days toseeking care,mean(SD)	6.6 (5.7)	3.6 (3.1)	6.3 (6.4)	4.5 (3.0)	5.3 (5.0)	7.8 (8.4)	2.6 (0.7)	4.9 (4.9)	5.2 (6.1)	5.3 (5.7)

DoD, Department of Defense; FRI, febrile respiratory illness; hMPV, human metapaneumovirus; RSV, respiratory syncytial virus; SARI, severe acute respiratory infection SOB, shortness of breath.

*SARI cases only identified from US-Mexico border populations.

**Table 2:** Age-stratified Descriptive Characteristics by Influenza, Other, and No/Unknown Pathogen among DoD Beneficiaries and US-Mexico Border Populations.

AgeGroup,Years	0–4 (n = 270)				5–24 (n = 290)				25+ (n = 259)			
Characteristic	Influenza(n = 38)	Other(n = 137)	No/UnknownPathogen(n = 95)	*P* value	Influenza(n = 82)	Other(n = 63)	No/UnknownPathogen(n = 145)	*P* value	Influenza(n = 48)	Other(n = 44)	No/UnknownPathogen(n = 167)	*P* value
Sex(% male)	19 (50)	66 (48)	48 (51)	0.94	42 (51)	27 (43)	61 (43)	0.42	17 (35)	19 (43)	73 (44)	0.57
Studypopulation				0.0071*				0.0061*				0.027*
US-Mexicoborder	23 (61)	76 (55)	35 (37)		43 (52)	26 (41)	45 (31)		21 (44)	24 (55)	108 (65)	
DoDbeneficiaries	15 (39)	61 (45)	60 (63)		39 (48)	37 (59)	100 (69)		27 (56)	20 (45)	59 (35)	
Severity				0.038*				0.11				0.0017*
Outpatient(FRI)	35 (92)	104 (76)	81 (85)		78 (95)	57 (90)	141 (97)		35 (73)	25 (57)	74 (44)	
Inpatient(SARI)*	3 (8)	33 (24)	14 (15)		4 (5)	6 (10)	4 (3)		13 (27)	19 (43)	93 (56)	
Signs andsymptoms												
Pneumonia	2 (6)	19 (15)	6 (7)	0.088	3 (4)	4 (7)	5 (4)	0.58	10 (22)	10 (24)	58 (36)	0.091
Sore throat	23 (61)	46 (34)	48 (51)	0.0023*	68 (83)	47 (75)	117 (81)	0.44	39 (81)	28 (64)	78 (47)	<0.0001*
Cough	37 (97)	132 (96)	86 (91)	0.20	79 (96)	58 (92)	120 (83)	0.0052*	47 (98)	40 (91)	149 (89)	0.17
Nausea	14 (37)	39 (28)	28 (29)	0.63	36 (44)	23 (37)	61 (42)	0.62	28 (58)	13 (30)	59 (36)	0.0066*
SOB	5 (13)	38 (28)	20 (21)	0.12	19 (23)	14 (22)	29 (20)	0.84	24 (50)	25 (57)	106 (63)	0.22
Congestion	29 (78)	88 (68)	68 (76)	0.29	64 (79)	47 (76)	93 (65)	0.057	37 (77)	26 (59)	72 (46)	0.0007*
Pink eye	1 (3)	8 (6)	7 (8)	0.56	11 (14)	3 (5)	8 (6)	0.058	6 (13)	1 (2)	2 (1)	0.0012*
Body ache	12 (32)	23 (17)	21 (22)	0.13	50 (62)	39 (62)	76 (52)	0.27	43 (90)	35 (80)	114 (68)	0.0080*
Headache	9 (24)	18 (13)	20 (21)	0.18	55 (68)	36 (57)	95 (66)	0.38	39 (81)	28 (64)	99 (60)	0.023*
Fever(≥100°C)	28 (74)	92 (67)	47 (49)	0.0066*	62 (76)	34 (54)	62 (43)	<0.0001*	20 (42)	18 (41)	77 (46)	0.76
Days toseekingcare, mean(SD)	3.6 (2.2)	4.3 (4.9)	3.7 (3.1)	0.43	2.9 (1.6)	4.1 (3.8)	4.0 (2.8)	0.0076*	4.7 (5.1)	5.3 (3.8)	6.2 (8.3)	0.38

DoD,Department of Defense; FRI, febrile respiratory illness; SARI, severe acute respiratory infection SOB, shortness of breath.

*SARI cases only identified from US-Mexico border populations.

**Table 3:** Descriptive Characteristics by Rhinovirus, Other, and No/Unknown Pathogen among Military Recruits (n = 615).

Characteristic	Rhinovirus (n = 173)	Other (n = 148)	No/Unknown (n = 294)	*P* value
Sex (% male)	156 (90)	127 (89)	235 (82)	0.024*
Signs and symptoms				
Pneumonia	95 (55)	50 (34)	116 (40)	0.0004*
Sore throat	143 (83)	115 (78)	237 (81)	0.53
Cough	163 (94)	144 (97)	234 (80)	<0.0001*
Nausea	68 (39)	46 (32)	108 (37)	0.33
SOB	104 (60)	60 (41)	142 (48)	0.0018*
Congestion	158 (91)	121 (82)	235 (80)	0.0055*
Pink eye	14 (8)	6 (4)	11 (4)	0.096
Body ache	96 (56)	89 (60)	194 (66)	0.074
Headache	128 (74)	110 (75)	230 (79)	0.48
Fever (≥100°C)	69 (40)	96 (65)	168 (57)	<0.0001*
Days to seekingcare, mean (SD)	7.8 (6.1)	6.0 (6.1)	5.8 (6.5)	0.0029*

SOB, shortness of breath.

Statistical analysis was conducted using SAS software (version 9.3, SAS Institute, Inc., Cary, North Carolina). PROC LOGISTIC with link = GLOGIT was used for the multinomial modeling and PROC ANOVA and PROC FREQ were used for ANOVA and chi-squared analyses, respectively.

## Results

During the 18 months of the study, October 2011 through March 2013, 1444 patients met the FRI or SARI case definitions and were enrolled in this study, consisting 406 (28%) from the FRI/SARI border, 423 (29%) from the DoD beneficiary, and 615 (43%) from the FRI recruit populations. The percent positivity for each pathogen out of all specimens tested was rhinoviruses (16%), influenza (14%), RSV (6%), adenovirus (2%), coronaviruses (5%), bacterial (2%), hMPV (1%), and coinfections (6%). Influenza A (H3N2) was the most common influenza subtype, making up 54% of influenza specimens, followed by influenza B (31%), and pH1N1 (13%). Among coronaviruses, CoVOC43 was the most commonly identified strain, making up 67% of coronavirus specimens, followed by CoV229E (21%) and CoVNL63 (12%). (Table 1) Additional testing found that 1% (8/571) of a subset of specimens were positive for parainfluenza viruses.

**Figure 1:**
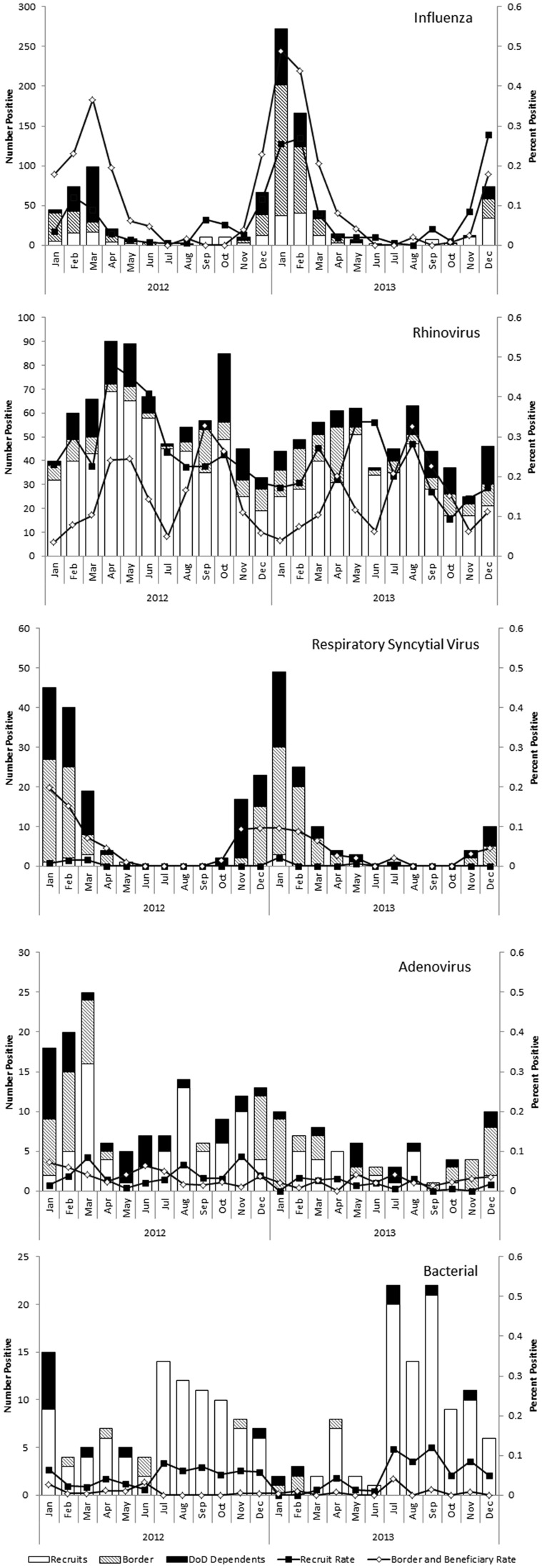
Number positive and percent of febrile respiratory illness/severe acute respiratory infection (FRI/SARI) patients positive for respiratory pathogens among US military recruits, DoD beneficiaries, and US–Mexico border populations, 2012–2013.

Seasonal patterns were apparent for influenza, rhinovirus, and RSV, whereas more consistent low levels of infection were seen for adenovirus and bacterial infections. The recruit population had a more constant number of FRI cases sampled than the other two populations throughout the study period. Overall, they also had a greater percent positivity for rhinovirus compared with the other two populations, but lower percent positivity for influenza and RSV. Rhinovirus appeared to have two peaks: one in spring and one in summer/fall among the border and beneficiary populations and consistently higher levels among the recruit population. In all populations influenza and RSV peaked in the winter (Fig. 1). Influenza A typically peaked before influenza B.

Rhinovirus and bacterial infections were more frequently isolated from recruits and men who make up the majority of the recruit population; whereas influenza and RSV were less frequent among these groups compared with the DoD beneficiary and border populations. Fewer rhinovirus and more RSV pathogens were identified from SARI cases compared to FRI. Pneumonia and shortness of breath were more frequent among rhinovirus cases and less common among influenza cases. However, time to seeking care was shorter for influenza and hMPV and longest for bacterial infections. Influenza, RSV, adenovirus, and hMPV cases all had higher temperatures compared with no/unknown pathogens, and rhinovirus has significantly lower temperature (Table 1).

Descriptive statistics for influenza, other, and no/unknown pathogen among DoD beneficiaries and US-Mexico border populations showed different clinical presentation across three age groups. Among 0–4 year olds, influenza was more frequent among US-Mexico border populations compared to DoD Beneficiaries. In the youngest age group, sore throat and fever were also more frequent among those positive for influenza compared to other or no/unknown pathogen and among 5–24 year olds, cough, fever, and short time to seeking care were more frequent. Finally, among the 25 and older age group, sore throat, nausea, congestion, pink eye, body ache, and head ache were more frequently seen in the influenza group compared to the other groups. Interestingly, fever was the least frequent among influenza positive participants in the oldest age group (Table 2).

Clinical presentation among recruits also differed by those with laboratory confirmed rhinovirus, other pathogen, or no/unknown pathogen. A higher percentage of males were diagnosed with rhinovirus or other pathogen compared to the other and no/unknown pathogen. Additionally, participants with rhinovirus had higher percentages of pneumonia, shortness of breath, congestion, and less fever and longer time to seeking care than the other groups and participants with either rhinovirus or other pathogen had higher cough than the no/unknown pathogen group (Table 3).

Building on the descriptive statistics, we found that fever was predicative of influenza compared to no/unknown pathogen among 0–4 year olds. Among 5–24 year olds, fever, cough, and short time to seeking care were predicative of influenza compared to no/unknown pathogen. Finally, among those 25 years and older, sore throat and nausea were predictive of influenza compared to no/unknown pathogen. (Table S1 in S1 File). Among the recruit population, cough and less fever were predictive of rhinovirus, and cough, fever, and less shortness of breath were predictive of other pathogen compared to no/unknown pathogen. (Table S2 in S1 File).

At least one pathogen was identified in 51% of all FRI and SARI cases. Coinfections were found in 81 (6%) of all FRI/SARI cases tested, among which were 76 double, four triple, and one quadruple coinfections. The most frequent pathogen associated with a coinfection was rhinovirus, followed by RSV and CoVOC43. The top three most common coinfections were rhinovirus and RSV (15% of all coinfections, 11 cases), rhinovirus and adenovirus (13% of all coinfections, 10 cases), and rhinovirus and CoVOC43 (12% of all coinfections, 9 cases). Interestingly, our study found an overall coinfection percent positivity of 6% which declined with age: 11% of 0–4 year olds, 5% of 5–24 year olds, 3% of 25–49 year olds and 2% of 50+ year olds. (Table 4).

**Table 4:** Detection of Respiratory Coinfections 2011–2013 (n = 1444).

	Rhinovirus	Influenza A	Influenza B	RSV	CoV229E	CoVOC43	CoVNL63	Adenovirus	hMPV	*M.* *pneumoniae*	*C.* *pneumoniae*	DetectionRate (%)
% coinfections	20	11	10	21	35	29	42	35	21	21	17	
Rhinovirus	**296**	6	5	11	3	9	4	10	3	3	1	20
Influenza A		**152**	0	3	1	1	1	1	0	1	0	11
Influenza B			**69**	2	0	0	0	0	0	0	0	5
RSV				**109**	1	4	0	0	0	0	0	8
CoV229E					**20**	0	0	0	0	0	0	1
CoVOC43						**65**	0	3	0	1	0	5
CoVNL63							**12**	0	0	0	0	8
Adenovirus								**51**	0	2	0	4
hMPV									**14**	0	0	1
*M. pneumoniae*										**34**	0	2
*C. pneumoniae*											**6**	0
1 virus	236	135	62	86	12	46	7	33	11	27	5	46
2 viruses	55	14	7	21	5	18	5	16	3	7	1	6
3 viruses	4	2	0	2	2	1	0	1	0	0	0	0
4 viruses	1	1	0	0	1	0	0	1	0	0	0	0

Four triple and one quadruple coinfections were identified but not included in upper portion of the table: rhinovirus/adenovirus/RSV, rhinovirus/influenza A/CoV229E, rhinovirus/influenza A/CoVOC43, rhinovirus/RSV/CoV229E, and rhinovirus/influenza A/adenovirus/RSV.

*C. pneumonia*, *Chlamydophila pneumoniae*; hMPV, human metapneumovirus; M. pneumoniae, *Mycoplasma pneumoniae*; RSV, respiratory syncytial virus.

## Discussion

In order to reduce respiratory disease burden, it is necessary to gain a better understanding of the percent positivity, coinfection rates, and seasonality of specific respiratory pathogens. Additionally, a predictive clinical model that uses symptoms, seasonality, and patient demographics can also help improve prevention efforts and patient treatment. Timeliness is especially important for influenza antivirals, which work best if given within the first 48 hours of symptoms. However, rapid diagnostic tests have poor sensitivity [23] and multiplex PCR tests are impractical for most clinical settings. Additionally, treatment with antibiotics can often be incorrectly prescribed for viral infections, leading to increased antibiotic resistance. Therefore, creating models to better predict the type of pathogen using symptoms and characteristics easily collected by a clinician at the time of visit could improve treatment accuracy and help protect the effectiveness of existing antibiotics and antivirals.

Many respiratory etiology studies have been done outside the United States among patients with both severe, lower respiratory illness [24], [25], [26], as well as more mild, upper respiratory disease [7], [8], [9], [10], [11], [12]. These studies are important because viral etiologies vary across populations and regions, depending on factors such as population susceptibility, age, circulating strains, climate, comorbidities, and vaccination coverage. Our study adds to the very limited number of etiology studies done in the United States [2], [3], [4] by examining viral etiology among three different US populations: military recruits, DoD dependents, and a US–Mexico border civilians and including all ages. Our study is also unique in that it collected clinical signs, symptoms, and demographics for each case tested with the broad-spectrum respiratory panel. Similar studies have been limited and have used smaller sample sizes, focused on one age group, and did not test for as many respiratory pathogens. Identifying population-specific baselines of infection enables us to identify elevated rates, which may indicate an outbreak or the start of a pandemic. Recognizing associated symptoms can help determine the most likely pathogen, as was seen in 2009 with the pandemic influenza (H1N1) strain first identified in Brawley and San Diego, California, in two of the populations in this study [27].

There were several key differences of infection among the three populations. The recruit population had consistently higher levels of rhinovirus and bacterial infections than the other groups, which may be reflective of close living conditions and a younger age group, mostly 18–24 years old. This population is also highly vaccinated for influenza and showed the smallest amount of influenza infection compared with the other two populations. The higher frequency of influenza among 0–4 year olds in the US-Mexico border population (61%) compared to beneficiaries (39%) may be reflective of different exposures or differences in vaccination uptake. However, the overall frequency of influenza found between the two groups was similar. Adenovirus, which historically had a large impact on recruits, was also low as a result of resumption of the adenovirus vaccines in October 2011 [28]. RSV, which usually infects young children, was more commonly found in the DoD dependent (48%) and border populations (48%) compared with the recruits (4%). Overall, rhinoviruses were the most common respiratory pathogen identified (236 cases, 16%), followed by influenza (197 cases, 14%), RSV (85 cases, 6%), and coronaviruses (66 cases, 5%), which coincides with other etiology studies [13].

The results of the age-stratified influenza, other pathogen, and no/unknown results showed some interesting differences across age groups. Most interesting was the relatively low percentage of influenza positive participants with fever (42%) in the 25 and older year age group, compared to the 0–4 (74%), and 5–25 year olds (76%). Fever may sometimes be masked by the consumption of antipyretics, although this group also complained of high headache (81%) and body ache (90%). The presentation differences of influenza by age could have some important implications for influenza surveillance, as the standard influenza-like illness and SARI case definitions require [29]. Consequently, an age-stratified case definition may be more appropriate as the standard case definitions may be underestimating the burden of influenza in older age groups. Circulating strains may also influence clinical presentation across age groups. (Table 2, Table S1 in S1 File).

In our 5–24 year olds we found fever, cough, and short time to seeking care to be predictive of influenza infection. Unfortunately, previous studies assessing the diagnostic accuracy of fever, cough, and acute onset to predict flu have found them to have low sensitivity (20%), and high specificity (96%) [18]. Another study, found that sore throat and fever in participants less than 5 years old had a sensitivity of 51% and specificity of 54% and cough and fever among those greater than 5 years had a sensitivity of 80% and specificity of 42% [30]. Although identifying predictive symptoms can be useful, it is important to recognize that diagnostic accuracy may still be low due to overlapping symptoms of many respiratory infections and may change due to fluctuations in circulating strains, age of the population and comorbidities. (Table 2, Table S1 in S1 File).

Rhinovirus was the most frequently identified pathogen among recruits, although it typically does not present with severe illness or acute onset, which is reflected in the long time to seeking care among rhinovirus positive participants. Interestingly, we also found the lowest percentage of fever among participants positive for rhinovirus compared to any other respiratory pathogen (Table 1). These findings are similar to other studies [13], [14], [31] and could have important implications for designing respiratory disease surveillance systems to capture outbreaks of viruses in the same family. For example, the recent outbreak of enterovirus 68 has similar symptoms of low fever [32]. (Table 3, Table S2 in S1 File).

Understanding coinfections can also be useful for preventing respiratory disease. Our study found around 30% to 40% of coronavirus and adenovirus infections occurred as coinfections and they most frequently occurred with rhinovirus. Similarly, other studies have also found the highest ratio of coinfections among adenovirus and coronaviruses [33], [34], and have found rhinovirus to be part of the most frequently occurring coinfections [26], [33]. These results suggest that infection with some viruses, such as rhinoviruses, could create opportunistic environments for colonization with other viruses and bacteria. Interestingly, coinfections were most common among the youngest age group, newborn to four years, which did not have the highest rhinovirus rate. Targeting rhinovirus infection through creation of new vaccines or treatment could have more far-reaching benefit in protecting a person from other infections.

Respiratory coinfection rates have varied across studies and are likely influenced by age, type of case definition used to enroll participants, and pathogens tested. Previous studies with a median age of 2–3 years old have found coinfections among SARI and acute respiratory tract infections of 17% and 19%, respectively [26], [33]. A third study among childcare attendees in the US found even higher coinfection rates of 47% [4]. Finally, a study from Scotland that tested respiratory specimens from all age groups found a coinfection rate of 5% [34]. These studies coincide with ours and show high coinfection rates among children. Declining coinfection rates with age may be a result of overall increased immunity to a broad range of respiratory pathogens as people get older.

One limitation of this study is that it only captured people with FRI/SARI who sought medical care. Military recruits may be less likely to seek care than other groups due to concern over losing training time or having to restart the program. Therefore, the etiology of more mild infections may be underrepresented for these two groups. Additionally, the case definitions were slightly different for the three populations, which may have influenced which pathogens were identified in each group. Although this study involved three different US populations, the results of this study may be less generalizable to the general US public who are not associated with the military or living on the US–Mexico border. Despite this, signs and symptoms from these pathogens should be similar across other populations in similar age groups and with similar vaccination coverage. Additionally, we found that seasonality of infection for recruits was similar to that of the border and beneficiary populations for several pathogens, but with different intensity. Consequently, illness surveillance in recruits, which made up the largest proportion of our study population, can be beneficial in informing disease trends in the general public.

Although we tested for many pathogens, there are likely still circulating viruses and bacteria for which we did not test, such as bocavirus, CoVHKU1, or potentially unrecognized viruses; therefore, these cases were likely incorrectly classified as part of the “no pathogen” group. Additionally, the timing of sample collection in the course of illness could impact whether or not viruses were identified by PCR. Symptom collection may also be biased in the youngest age group of 0–4 year olds, who may not be able to articulate how they are feeling. Despite this, our study was part of a well-established existing surveillance program that consistently collected and tested a substantial number of specimens at many different sites across the United States. In the future, additional years of surveillance data will continue to improve our understanding of seasonality.

Although nothing will replace the accuracy of laboratory diagnostics, a broader understanding of seasonality, clinical presentation, demographics, and coinfection rates of pathogen specific respiratory diseases can improve diagnosis and treatment, by informing clinicians on appropriate antiviral and antibiotic treatment during the patient’s visit. This can ultimately reduce the number of lost work days and transmission. Additionally, describing baseline of disease and seasonality for specific pathogens can improve our ability to detect outbreaks. Identifying the percent positivity of each pathogen, coinfection rates, and risk factors for disease will help inform vaccination programs, and possible investment in the development of future vaccines or treatments.

## Supporting Information

S1 File **Tables S1 and S2. Table S1.** Age Stratified Multinomial Logistic Regression Predicting Influenza, Other Pathogen, or No/Unknown Pathogen among DoD Beneficiaries and US-Mexico Border Populations Using Participant Characteristics. **Table S2.** Multinomial Logistic Regression Predicting Rhinovirus, Other Pathogen, or No/Unknown Pathogen among Military Recruits Using Participant Characteristics, Recruits, N = 613.
(DOCX)
