# Supplementary material for: Epidemiology of Pathogen-Specific Respiratory Infections among Three US Populations
Source: PLoS One. 2014 Dec 30;9(12):e114871. doi: 10.1371/journal.pone.0114871 (PMC4280218; doi:10.1371/journal.pone.0114871)
Supplement: S1 File — Tables S1 and S2. Table S1. Age Stratified Multinomial Logistic Regression Predicting Influenza, Other Pathogen, or No/Unknown Pathogen among DoD Beneficiaries and US-Mexico Border Populations Using Participant Characteristics. Table S2. Multinomial Logistic Regression Predicting Rhinovirus, Other Pathogen, or No/Unknown Pathogen among Military Recruits Using Participant Characteristics, Recruits, N = 613. (DOCX) [file pone.0114871.s001.docx]

**Table S1.**

|  |  | Influenza | | Other | | | | |  |
| --- | --- | --- | --- | --- | --- | --- | --- | --- | --- |
| Age Group | Characteristic | OR | 95% CI | OR | 95% CI | *P* Value | | |  |
| 0-4 year olds (n=269) |  |  |  |  |  |  | | |  |
|  | Sore throat | 1.35 | 0.62-2.94 | 0.45 | 0.26-0.79 | 0.0020 | | |  |
|  | Fever | 2.73 | 1.19-6.26 | 2.19 | 1.26-3.80 | 0.0069 | | |  |
| 5-24 year olds (n=290) |  |  |  |  |  | |  |  |  |
|  | Cough | 7.00 | 1.96-24.99 | 2.38 | 0.86-6.63 | 0.0055 | | |  |
|  | Fever | 3.70 | 1.95-7.02 | 1.63 | 0.87-3.07 | 0.0003 | | |  |
|  | Days to seeking care | 0.86 | 0.71-0.97 | 1.01 | 0.91-1.12 | 0.046 | | |  |
| 25 + year olds (n=258) |  |  |  |  |  |  | | |  |
|  | Sore throat | 4.56 | 2.06-10.10 | 2.10 | 1.05-4.20 | 0.0003 | | |  |
|  | Nausea | 2.16 | 1.09-4.26 | 0.70 | 0.33-1.45 | 0.026 | | |  |

**Table S2.**

|  | Rhinovirus  (n=173) | | Other  (n=148) | | |  |
| --- | --- | --- | --- | --- | --- | --- |
| Characteristic | OR | 95% CI | OR | 95% CI | *P* Value |  |
| Cough | 2.67 | 1.26-5.65 | 12.62 | 4.36-36.54 | <0.0001 |  |
| Congestion | 1.86 | 0.99-3.52 | 0.76 | 0.44-1.33 | 0.043 |  |
| Shortness of breath | 1.15 | 0.77-1.75 | 0.61 | 0.40-0.94 | 0.020 |  |
| Fever | 0.60 | 0.40-0.89 | 1.53 | 0.99-2.35 | 0.0004 |  |
